# Similar perspectives on prostate cancer screening value and new guidelines across patient demographic and PSA level subgroups: A qualitative study

**DOI:** 10.1111/hex.12517

**Published:** 2016-11-02

**Authors:** Melissa R. Partin, Sarah E. Lillie, Katie M. White, Timothy J. Wilt, Kristin L. Chrouser, Brent C. Taylor, Diana J. Burgess

**Affiliations:** ^1^ Center for Chronic Disease Outcomes Research (CCDOR) Minneapolis Veterans Affairs Health Care System Minneapolis MN USA; ^2^ Department of Medicine University of Minnesota Minneapolis MN USA; ^3^ School of Public Health University of Minnesota 1 Veterans Drive Minneapolis MN USA; ^4^ Department of Urology University of Minnesota Minneapolis MN USA; ^5^ Minneapolis Veterans Affairs Health Care System Minneapolis MN USA

**Keywords:** cancer screening, patient education, practice guideline, prostate cancer, qualitative research, race

## Abstract

**Background:**

In 2012, the United States Preventive Services Task Force (USPSTF) recommended against prostate‐specific antigen (PSA)‐based prostate cancer screening for all men.

**Objective:**

To inform educational materials addressing patient questions and concerns about the 2012 USPSTF guidelines, we sought to: (i) characterize patient perceptions about prostate cancer screening benefits, harms and recommendations against screening, and (ii) compare perceptions across race, age and PSA level subgroups.

**Methods:**

We conducted qualitative interviews with a sample of 26 men from the Minneapolis Veterans Affairs Health Care System, stratified by race (African American, other), age (50‐69, 70‐84) and PSA level (documented PSA level ≥4 in Veterans Health Administration electronic medical records vs no such documentation). We used an inductive approach informed by grounded theory to analyse transcribed interviews.

**Results:**

Most men in all subgroups expressed misperceptions about the benefits of prostate cancer screening and had difficulty identifying harms associated with screening. In all subgroups, reactions to recommendations against screening ranged from unconditionally receptive to highly resistant. Some men in every subgroup initially resistant to the idea said they would accept a recommendation to discontinue screening from their provider.

**Conclusions:**

Given the similarity of perceptions and reactions across subgroups, materials targeted by race, age and PSA level may not be necessary. Efforts to inform decision making about prostate cancer screening should address misperceptions about benefits and lack of awareness of harms. Provider perspectives and recommendations may play a pivotal role in shaping patient reactions to new guidelines.

## 1 Introduction

Because cancer screening is one important approach to reducing cancer‐related morbidity and mortality, decades of effort have been exerted explicitly to promote cancer screening behaviours. However, there is a growing appreciation of the potential harms associated with screening and the fact that the overall balance of benefits vs harms (ie screening value) may be less pronounced than originally thought.^1^, ^2^ In particular, there is increasing recognition that for some (especially the elderly or those with life‐limiting comorbidities), the harms of screening outweigh the benefits.^3^ Concerns about the unfavourable balance of benefits and harms are particularly pronounced for prostate cancer screening. In its 2012 recommendation statement for prostate cancer screening, the United States Preventive Services Task Force (USPSTF) recommended against prostate‐specific antigen (PSA)‐based prostate cancer screening for all men, because it concluded from the available evidence that the very low probability of preventing a death from prostate cancer in the long term (1 in 1000 men screened or less) does not outweigh the moderate‐to‐high probability of early and persistent harms.^4^ The harms of PSA screening and subsequent diagnostic tests and treatments can be serious and include the following: anxiety about test results,^5^, ^6^ hospitalizations resulting primarily from resistant E. Coli infections following trans‐rectal prostate biopsies (experienced by 1‐2 in 1000 men screened);^4^, ^7^ serious cardiovascular events following prostatectomy (experienced by 3 in 1000 men screened);^4^ and erectile, urinary and bowel dysfunction following surgery and other prostate cancer treatments (experienced by 35 in 1000 men screened).^8^ The USPSTF statement recommending against PSA screening spurred considerable debate among experts,^9^, ^10^, ^11^, ^12^ and a nationally representative survey of men aged 40‐74 conducted shortly after the draft recommendations were published found that, while the majority (62%) agreed with the recommendation, few (13%) intended to follow it.^13^

Despite the controversy surrounding the USPSTF prostate cancer screening recommendations, national data from the year following their release documenting modest but statistically significant declines in PSA screening in every age group suggest that at least some patients and providers are receptive to discontinuing screening.^14^, ^15^ Furthermore, the recommendations have been incorporated into clinical practice guidelines in some U.S. integrated health‐care systems, including the Veterans Health Administration (VHA). In 2013, the VHA issued a clinical practice guidance statement recommending against PSA screening in average‐risk men of any age and high‐risk men (ie African American men and men with a family history of prostate cancer) age less than 45 or greater than 69. For higher‐risk men aged 45‐69, the VHA endorses shared decision making about PSA screening. In anticipation of the need to address patient questions and concerns about its new prostate cancer screening guidance, the VHA requested our collaboration in preparing patient‐directed educational materials summarizing the updated evidence on the benefits and harms of prostate cancer screening, to be disseminated with the VHA guidance statement.

To inform the content of these materials, we sought information on patient perspectives about the value of prostate cancer screening and reactions to new guidelines recommending against PSA screening. Recent studies conducted in settings outside of the VHA suggest that patients may have inaccurate perceptions about the benefits and harms of screening tests,^16^ including prostate cancer screening,^17^, ^18^ and that some patient subgroups (including African Americans) may be less receptive to following USPSTF prostate cancer screening guidelines.^13^ However, it is not clear whether these findings, derived from studies conducted in men age if 50‐74, extend to other subgroups, including those unlikely to benefit from prostate cancer screening (men of any race over age 74), those with prior elevated PSA test results and those receiving care from the VHA. Older men and those with a history of elevated PSA results are particularly important subgroups to examine, because, given their prior experience with PSA screening, they may be more invested in the idea that PSA screening is helpful and accordingly more resistant to changing their screening practices. Given the lack of information on the perspectives of these subgroups in the existing literature, we conducted a qualitative study among Veterans receiving care in the VHA to characterize patient perceptions about prostate cancer screening benefits, harms and new guidelines recommending against PSA prostate cancer screening. To explore whether targeted materials might be warranted for patients with differing prostate cancer risk profiles, chance of benefitting from PSA screening or prior experience with positive screening, we compared perceptions and reactions expressed in different race (African American, other), age (50‐69, 70‐84) and PSA level (prior elevated result, no prior elevated result) subgroups. Our choice of these stratifying characteristics was informed by the Preventive Health Model.^19^ This model posits that background factors (such as demographics, medical history and prior health behaviours) can influence preventive health behaviours and intentions through their association with behavioural beliefs (such as perceived severity and susceptibility to the disease, worry, and perceived social norms and influences).

## 2 Methods

### 2.1 Design and participants

We conducted semi‐structured, individual, in‐person qualitative interviews in June 2013 with patients receiving care at the Minneapolis Veterans Affairs Health Care System (MVAHCS). Eligible participants, identified from VHA electronic medical records, included the following: male Veterans aged 50‐84; without a diagnosis of prostate cancer or dementia; who attended at least one outpatient visit with a MVAHCS primary care provider in the past year; who received a PSA from any VHA facility in the past 2 years; and who had complete address and phone information available. We excluded nursing home and adult day care residents, non‐English speakers and deceased individuals from our sample prior to recruitment.

### 2.2 Sample

We used purposive sampling to include patients representing different demographic and screening experience subgroups. Specifically, we stratified our study sample by race (African American; non‐African American), age (50‐69; 70‐84) and PSA level (documentation of a PSA level ≥4 in VHA electronic medical records vs no such documentation), as shown in Table 1.

**Table 1:** Number eligible, sampled, recruited and interviewed by sampling strata and subgroup of interest

Strata		Subgroup			Eligible	Sampled	Recruited	Interviewed
A	African Americans with elevated PSA age 50‐69	1	3	5	61	25	4	4
	African Americans with elevated PSA age 70‐85			6			1	1
B	African Americans without elevated PSA age 50‐69		4	5	633	25	4	4
	African Americans without elevated PSA age 70‐85			6			1	1
C	Non‐African Americans with elevated PSA age 50‐69	2	3	5	574	25	5	4
D	Non‐African Americans with elevated PSA age 70‐85			6	7795	25	5	4
E	Non‐African Americans without elevated PSA age 50‐69		4	5	400	25	5	4
F	Non‐African Americans without elevated PSA age 70‐85			6	2080	25	5	4
**TOTAL**					11 543	150	30	26

Subgroup		Strata	Eligible	Sampled	Recruited	Interviewed
1	African Americans	A, B	695	50	10	10
2	Non‐African Americans	C, D, E, F	10 848	100	20	16
3	Elevated PSA	A, C, D	8430	75	15	13
4	Non‐elevated PSA	B, E, F	3113	75	15	13
5	Age 50‐69	A – F	11 543	150	18	16
6	Age 70‐85				12	10

From the 11 543 patients meeting eligibility criteria, we randomly selected 150 to recruit to the individual interviews (25 per strata in Table 1). We mailed these 150 eligible individuals a letter describing the study, and alerting them to the fact that a VA employee might call them in the next few weeks to see whether they would be interested in participating in an interview. The letter included a phone number and email address to contact whether the Veteran preferred not to be called. We then proceeded to call Veterans who did not opt out of the study (n=150), until we recruited five participants within each stratum (or 30 overall). We scheduled Veterans agreeing to participate for one‐hour individual interview slots. We offered Veterans who completed interviews $40 compensation for their time and travel expenses.

Because we used our findings to develop educational materials needed within a constrained timeline, we determined sample size a priori, based both on expert recommendations for minimum qualitative sample sizes^20^ and on resource availability within the pre‐specified timeline. Based on the findings of one prior study documenting that 90% of high‐frequency themes were identified after six interviews,^21^ we sought to obtain 6‐10 completed interviews in each subgroup of interest: age (50‐59, 70‐84), race (African American, non‐African American) and PSA level (elevated, not elevated). We completed 10‐16 interviews in each of these subgroups and conducted post hoc descriptive analyses of code saturation (described below) to assess whether these sample sizes were sufficient to identify high‐frequency themes. We did not stratify the African American sample by age due to the small number of African American men over the age of 70 in the sampling frame, but did complete interviews with two African American men aged 70‐84.

### 2.3 Data collection

All interviews were conducted in‐person in a private interview room at the MVAHCS. Each individual interview was attended by two study staff: one investigator with qualitative research experience who asked the interview questions, and another team member who took notes and handled the recording equipment, consents and payment forms. The interview questions focused on what men knew about prostate cancer and the PSA test, what they thought the benefits and harms of screening were and how they would react if their provider recommended they not be tested (see interview guide, Appendix S1). Participants were not provided information on the benefits and harms of prostate cancer screening or new recommendations before the interview.

### 2.4 Analysis

We recorded and transcribed all interviews and imported the transcribed data into qualitative software (NVIVO 10) for coding and analysis. We did not return transcripts to participants for comment or correction. Our inductive analysis approach was informed by grounded theory^22^ (ie codes and themes were identified emergently from the text rather than applied from pre‐existing frameworks or theories). We used this inductive approach rather than a deductive approach applying codes from the Preventive Health Model or other framework because, at the time the study was conducted, little was known about men's perceptions regarding prostate cancer screening benefits, harms, or new recommendations against screening. Our objective was therefore to characterize these perceptions to inform hypotheses to test in future quantitative studies. Our inductive coding and analysis approach involved several steps. In the first step, one investigator developed a provisional codebook based on the interview guide. In the second step, this investigator and a second coder independently reviewed transcripts in batches of 5‐8. For each batch, coders noted concepts emerging from the text, met to discuss emergent concepts and agree on conceptual categories, added agreed upon categories and examples to an evolving codebook, and then applied the codebook to the transcripts reviewed (see Table 2 for final coding tree). To facilitate the post hoc saturation analyses described below, we coded transcripts in the order in which the interviews were completed. In the third step, the two coders used the coding comparison feature in NVIVO to identify coding discrepancies, and then adjudicated all differences to arrive at final coding decisions for analysis. In the last step, the coders identified major themes and then compared the range and frequency of these themes across race, age and PSA level subgroups.

**Table 2:** Final coding tree

**Perceptions about benefits** Physical benefits Early detection / prevention of disease or disease spread Living longer Cure Small benefit Knowing / decision making Psychological benefits Being proactive / doing something **Perceptions about harms** No harms Physical harms Bleeding Pain Infection Impotence Problems with urination Psychological harms False positives False negatives Wasted resources – cost to system Financial harms – cost to individual Overdiagnosis **Reactions to guidelines** Receptivity Unconditional acceptance Conditional acceptance Trust in research Resistance Distrust – cost as motivator Scepticism and counter arguing Discomfort doing nothing Still prefer screening Trust provider Depends on context Uncertainty Want more information

To provide insights into potential theme saturation, we examined the number and per cent of all themes and high‐frequency themes (mentioned by more than 25% of the sample) included in our final codebook that were identified after each batch of interviews coded.

The study protocol was reviewed and approved by the Institutional Review Boards at the Minneapolis VA Health Care System and the University of Minnesota.

## 3 Results

### 3.1 Participant demographic characteristics

We scheduled interviews with 30 individuals and completed interviews with 26. The average interview length was 23 minutes (range 13‐47 minutes). Individuals completing interviews included 10 African American men, 16 non‐African American men, 16 men aged 50‐69, 10 men aged 70‐85 (five aged 70‐75 and five aged 76‐85), 13 men with a prior elevated PSA and 13 men without a prior elevated PSA (Table 3).

**Table 3:** Participant demographic characteristics

Characteristic	Number	%
Race		
African American	10	38
Non‐African American	16	62
Age (mean)	(67)	
50‐69	16	62
70‐75	5	19
76‐85	5	19
Prior PSA level ≥4		
Yes	13	50
No	13	50

### 3.2 Perceptions about benefits

When asked their perspectives on the potential benefits of screening, most men mentioned a physical benefit. The most common physical benefit mentioned in every subgroup was early detection and/or prevention of disease progression.

“It's like playing offense, you discover it in advance and you can do something about it.” (ID #1016 – non‐African American, 70‐85, without prior elevated PSA)

“Well, it might stop it or delay it so that you have a possible chance of not getting it full blown or worse.” (ID #1077 – African American, 50‐69, with prior elevated PSA)

Six to seven men in every subgroup expressed a belief that having a PSA will help you live longer.

“I would say you've got a 100% chance of living longer… the earlier you can detect it, the better your odds….that's the bottom line right there.” (ID# 1014 – Non‐African American, 50‐69, with prior elevated PSA)

“If you catch it in time or something you can probably extend your life.” (ID #1045 – African American, 70‐85, with prior elevated PSA)

At least two men in every subgroup mentioned cure as a benefit.

“By catching it early, you may be treated and not prolong the effects. Maybe have a chance of getting rid of it.” (ID #1036 ‐ African American, 50‐69, with prior elevated PSA)

“I'm thinking the benefit would be that if you catch it early you can eliminate prostate cancer.” (ID #1041‐ non‐African American, 70‐85, with prior elevated PSA).

One individual mentioned that the survival benefit is small.

“They're saying even if they do a total removal, it just doesn't extend it that much… maybe another month. It's not much.” (ID #1003 ‐ non‐African American, 50‐69, without prior elevated PSA)

After physical benefits, the most common benefit of PSA screening mentioned by men was “knowing or decision making.” This benefit was mentioned by most men in every subgroup.

“I'd rather have the test done and find out what's going on. At least I'll know. Then I'll make that decision.” (ID #1006 – African American, 50‐69, without prior elevated PSA)

“You can't manage what you don't know, and truly this is a data point.” (ID #1091 – non‐African American, 70‐85, with prior elevated PSA)

At least three men in every subgroup mentioned psychological benefits associated with a normal screening or biopsy result.

“From a psychological standpoint, once you know you're clean…I think you get on with your life and…you sleep better.” (ID #1016 – non‐African American, 70‐85, without prior elevated PSA)

“Knowing is relief…so you don't have to worry or wonder if I have it.” (ID #1036 – African American, 50‐69, with prior elevated PSA)

Finally, a few non‐African American men from both age groups without a prior elevated PSA mentioned being proactive or doing something to stay on top of one's health as a benefit of screening.

“It's kind of an important thing to me to protect myself and what's going on here.” (ID #1019 ‐ non‐African American, 50‐69, without prior elevated PSA)

### 3.3 Perceptions about harms

When asked what possible harms could result from PSA screening, the first response provided by most men in every subgroup was “none.”

“It's a blood test…they do blood draw for thousands of things and so what harm could that be?” (ID #1022‐ African American, 50‐69, with prior elevated PSA)

“I don't see any harm in having a clue…that something might be wrong and then you look at it with other tools” (ID #1001 ‐ non‐African American, 70‐85, without prior elevated PSA)

After prompting to consider complications of biopsy and prostate cancer treatment, at least seven men in every subgroup (including some that originally could not think of any harms associated with PSA screening) mentioned a possible physical harm associated with these downstream stages in the screening cascade, including bleeding, pain, infection, impotence or problems with urination.

At least two men in every subgroup mentioned psychological harms resulting from bad news or unfortunate side‐effects as a possible harm of PSA screening.

“Well, if it were proved positive you would go into depression.” (ID #1055 –African American, 50‐69, without prior elevated PSA)

“Sometimes a man can go into deep depression, because, you know, what they call impotence.” (ID #1004 – non‐African American, 70‐85, without prior elevated PSA)

At least one man in every subgroup mentioned false positives as a possible harm of PSA screening, and one non‐African American aged 70‐85 without a prior elevated PSA mentioned wasted resources – or financial costs to the system. Few mentioned other harms identified by experts^23^ and guideline‐issuing bodies, such as false negatives^24^; overdiagnosis and overtreatment;^4^, ^23^, ^24^, ^25^, ^26^ or financial costs to the individual.^23^

### 3.4 Reactions to recommendations against screening

When asked what they thought about new guidelines recommending against prostate cancer screening, at least five men in every subgroup expressed unconditional receptivity to the idea of discontinuing PSA.

“I think that it's a step in the right direction, really. There's no hesitation at all on my part to accept this, and I think it just makes a lot of sense.”(ID #1041 –non‐African American, 70‐85, with prior elevated PSA)

“I asked the last time I was out here did they do it. They generally said no. So you know if that's the case, that's fine.” (ID #1003 – African American, 50‐69, without prior elevated PSA)

A couple of men aged 50‐69 (one African American without a prior elevated PSA and one non‐African American with a prior elevated PSA) said they were comfortable with the new guidelines because of their trust in research.

“This latest recommendation …I did go back and take a look at it. It's pretty rigorous. It was based on pretty rigorous review… From a broad public health perspective they're probably right that this doesn't make sense.” (ID #1091 – non‐African American, 50‐69, with a prior elevated PSA)

However, most men in every subgroup expressed some resistance to the idea of forgoing screening, including the following:

#### 3.4.1 Distrust, or suspecting cost as a motivator

“You hear a lot about that now, that they're not going to screen as much… That people will be denied…it's such a slow growth cancer, should we have to pay for that.” (ID #1018‐ African American, 70‐85, without prior elevated PSA)

“They're trying to cost cut there or something like that and rewrite some of the criteria, I think.” (1082 – non‐African American, 50‐69, with prior elevated)

#### 3.4.2 Discomfort doing nothing

“There has to be something. I wouldn't want to leave out my testing…because there's no way she could tell me everything was all right if I wasn't tested.” (ID #1047 – African American, 50‐69, without prior elevated PSA)

“Then what are they going to replace this with? See, they gotta' replace it with something.” (ID #1074 – non‐African American, 70‐85, with prior elevated PSA)

#### 3.4.3 Prefer to continue screening

“Until the professional community straightens this thing out, I would say go ahead and have it.” (ID #1091 – non‐African American, 70‐85, with prior elevated PSA)

“I probably would ask for one anyway. When I go in, I'm likely to get everything tested.” (ID #1047 ‐ African American, 50‐69, without prior elevated PSA)

#### 3.4.4 Scepticism and counter arguing

“Okay, so the provider decides that you don't need this test, and then you die of prostate cancer; then what?” (ID #1006 – African American, 50‐69, without prior elevated PSA)

“I don't understand that at all. Why is this test causing a problem? A test is just blood, as far as I know.” (ID #1074 – non‐African American, 70‐85, with prior elevated PSA)

A few men expressing initial scepticism reacted so strongly to the idea of a provider recommending against screening that they said they would seek a different doctor.

“That is so ignorant that I cannot believe some doctors would be that crazy…and I'd tell a doctor right to his face ‘if you believe that … I don't want you to be my doctor.’” (ID #1045 –African American, 70‐85, with prior elevated PSA)

“I wouldn't want him to be my doctor or my primary care doctor.” (ID #1082 ‐ non‐African American, 50‐69, with elevated PSA)

However, four to five men in every subgroup who initially expressed resistance to the idea of stopping screening changed their response when asked how they would respond if *their own doctor* recommended they discontinue, expressing trust in their provider's recommendations. For example, when initially asked what they thought of new guidelines recommending against screening, two men said:

“I really can't be in favor of them saying don't have it. I really can't, because I think that's a silent killer and how you gonna know unless you take the test?” (ID #1016 –non‐African American, 70‐85, without prior elevated PSA)

“That's kind of confusing to me, because if it's a PSA, if it's a blood test…I've done blood draw for other things, and there was no problem. So I don't understand the risk of it.” (ID #1022 – African American, 50‐69, with elevated PSA)

When later asked how they would react if their own provider recommended they stop screening, these same men said:

“I'd probably want to indulge in more questions. But then if they gave me an answer, because I have faith and confidence in their decision‐making process, I'd accept that. I really would.” (ID #1016 –non‐African American, 70‐85, without prior elevated PSA)

Well, when she said I should have it, I said okay, and if she says, well, maybe you shouldn't have it, I'd just say okay, and that's it. (ID #1022 – African American, 50‐69, with elevated PSA)

At least four men in every subgroup said they would want more information about the harms before deciding whether to stop screening.

“If you could lay out the harms maybe I would say oh, jeez I never thought about that.” (ID #1001, non‐African American, 70‐85, without prior elevated PSA)

“What is the harm? I would want him to explain that.” (ID #1036 – African American, 50‐69, with prior elevated PSA)

One to four men in every subgroup said their reaction to a recommendation to discontinue screening would depend on the context, including their age or symptoms at the time of the recommendation.

“If I'm 85 years old…well, fine…the chances are I'm not gonna be around 20 years after that anyway to worry about it.” (ID #1017 – non‐African American, 50‐69, with prior elevated PSA)

“I guess I would have to weigh it in terms of how it's affecting me at the moment…if I was having a problem then I definitely would need to be tested, but if I don't feel that I was having a problem then I could avoid the test. (ID #1077 – African American, 50‐69, with elevated PSA)

Finally, a couple of younger men and men with a prior elevated PSA expressed uncertainty about their reactions to the new guidelines, but no older men or men without a prior elevated PSA expressed uncertainty.

The above difference in expressed uncertainty aside, our results suggest that perceptions and reactions were similar across all subgroups examined. All high‐frequency themes (mentioned by at least 25% of participants) were mentioned by every subgroup.

### 3.5 Saturation analysis

Our final codebook (Table 2) included 37 unique codes. Our saturation analysis revealed that we identified 92% of all codes and 100% of high‐frequency codes (ie those mentioned by more than 25% of participants) in the first five interviews completed. The only codes not identified in the first five interviews were two low‐frequency codes (mentioned by less than 25% of participants): small benefit and harms/physical/problems with urination.

## 4 Discussion

This study contributes to the literature on men's perceptions about prostate cancer screening value and reactions to new guidelines recommending against PSA by comparing perspectives and reactions across race, age and PSA level subgroups. An unanticipated finding of our work was the remarkable similarities in perceptions and reactions across these subgroups. This finding suggests that targeting materials by race, age and PSA level is likely not warranted in this population, which greatly simplifies implementation of decision support for the new VHA guidance statement. At least one prior qualitative study, conducted among unaffected first‐degree relatives of prostate cancer patients, similarly found no evidence to support race‐targeted materials for prostate cancer screening.^27^

While materials targeted by race, age or PSA level may not be necessary, materials that address the misperceptions about benefits and lack of awareness of potential harms expressed in all subgroups are clearly needed. Although PSA‐based prostate cancer screening has not been shown to reduce overall mortality, and any reductions in prostate cancer specific mortality are judged to be small (1 in 1000 or less) through at least 10‐15 years,^4^ some men in every subgroup thought PSA screening could help them live longer and perceived the chance of experiencing this benefit to be high. Only one individual in our sample seemed aware that the mortality reduction from PSA screening was small. Additionally, we found that most men in every subgroup had difficulty connecting PSA screening with any harms. These findings are consistent with results from prior qualitative and quantitative studies finding that the public tends to overestimate the benefits and underestimate the harms of screening tests generally,^16^ and prostate cancer screening specifically.^17^, ^18^ Further, recent studies suggest that primary care physicians also have inaccurate perceptions about cancer screening benefits and harms,^28^ which may contribute to misperceptions among patients.

We found that psychological benefits and harms associated with PSA results, which are rarely mentioned in cancer screening guidelines or decision aids, were important to men. This finding is consistent with findings from one prior qualitative study exploring patient perceptions of the benefits and harms of overused cancer screening tests.^17^ The paucity of information on the psychological benefits and harms in cancer screening decision aids may reflect a scarcity of data on their prevalence, a lack of appreciation of their importance by experts, or both. At least one group of experts has called for greater examination of potential psychological harms,^23^ and future research should seek to better quantify them for specific cancer screening tests.

Although not explicitly recognized as a benefit by experts^23^ or guideline‐issuing bodies,^4^ the majority of men in all subgroups considered knowing whether they have cancer as a benefit of PSA screening. At least two recent studies have noted this same perspective that more information is always better,^17^, ^29^ which may present a barrier to future efforts to reduce overdiagnosis and overtreatment associated with cancer screening.

We found that reactions to new recommendations against PSA screening ranged from unconditional receptivity to highly resistant. Prior research suggests that providers believe their patients expect them to offer PSA screening,^30^ and providers may be particularly wary of recommending against screening in patient subgroups at higher risk for prostate cancer or with prior elevated PSA test results. However, we found no evidence that men with higher risk of developing prostate cancer (African American men), greater likelihood of benefitting from PSA screening (younger men) or some experience with prior elevated PSA test results were more resistant to the idea of discontinuing PSA screening than other men. Further, some men initially expressing resistance to the idea of discontinuing PSA screening said they would willingly accept a recommendation to stop screening from a trusted physician, or someone who provided more information on the potential harms of screening to support such a recommendation. This finding underscores the well‐documented critical role that physician recommendations play in shaping patients PSA screening attitudes and behaviours.^31^, ^32^, ^33^, ^34^, ^35^

Our study has a number of strengths, including the rigorous coding approach, saturation analysis and comparison of perceptions and reactions across race, age and prior PSA result subgroups. However, our findings should be qualified by the following limitations. First, because this was a qualitative study, we cannot determine whether the prevalence or salience of certain perceptions and reactions varies significantly across the demographic and prior experience subgroups included in our study. Second, because our sample size was determined a priori rather than by saturation, we cannot be certain that all key themes were identified in our sample of 26 individuals. However, the findings from our post hoc saturation analysis provide reassurance that we likely identified all high‐frequency themes with the design employed. Third, we did not stratify our sample by prostate cancer family history, which the Preventive Health Model suggests could be associated with cancer screening attitudes and behaviours. However, evidence to support this association is mixed in the literature. While two prior studies have documented associations between prostate cancer family history, perceived prostate cancer risk (ie susceptibility) and prostate cancer screening behaviour,^36^, ^37^ one recent study found no association between prostate cancer family history and prostate cancer screening behaviour.^38^ Finally, because the VHA is a unique context, our findings may not generalize to other settings and populations. As the VHA is the largest integrated health‐care system in the United States, however, our findings have important implications for a substantial fraction of men in the United States.

## 5 Conclusions

Our findings suggest a similar range of reactions to new prostate cancer screening guidelines regardless of race, age or prior PSA screening. All of these subgroups expressed misperceptions about the benefits of PSA screening and difficulty understanding the connection between PSA screening and the downstream harms associated with prostate biopsies and prostate cancer treatment. Correcting these misperceptions will likely be a critical component of any efforts to implement evidence‐based clinical practice guidelines for prostate cancer screening, facilitate shared decision making or reduce prostate cancer overdiagnosis and overtreatment. Personal recommendations from trusted physicians to discontinue PSA screening may moderate initial discomfort with the idea of discontinuation. Future quantitative research should estimate the prevalence of misperceptions about prostate cancer screening benefits and harms in race, age and PSA level subgroups; test whether motivation to discontinue screening varies significantly across these subgroups; and quantify the effect of a physician recommendation to discontinue PSA screening on future screening intentions.
