# Supplementary material for: Similar perspectives on prostate cancer screening value and new guidelines across patient demographic and PSA level subgroups: A qualitative study
Source: Health Expect. 2016 Nov 2;20(4):779–87. doi: 10.1111/hex.12517 (PMC5513007; doi:10.1111/hex.12517)
Supplement: Supplementary file 1 [file HEX-20-779-s001.docx]

**Appendix 1: Interview guide**

**Introduction.**

- Thank you for agreeing to participate in this interview.
- Let’s start by discussing your opinions about prostate cancer and tests for prostate cancer.

**Prostate cancer knowledge and attitudes**

1. What have you heard or read about prostate cancer?

- Have you seen anything in the paper or on TV about it?

If yes:

- - What have you heard?
  - What do you think about that?
- Have you ever known someone who was diagnosed with prostate cancer?
  - Can you tell me more about that?
  - What have you heard about their experience?

1. Have you heard or read anything about screening tests for prostate cancer?

- Have you heard about the Prostate Specific Antigen, or PSA test? (if NO: the PSA test is a blood test to screen for prostate cancer).

1. Every cancer screening test has benefits and harms. What are the benefits of the PSA test for prostate cancer?

- How did you learn about these benefits?
- Have you or anyone you know benefited from testing?
- What were the benefits?
- What stories have you heard about people who have benefitted from prostate cancer screening?

1. What are the harms?

- How did you learn about these harms?
- What are the harms of follow-up tests that might be needed if the PSA test is elevated?
  - Have you or anyone you know had problems with follow-up tests like a prostate biopsy?
  - What were the problems?
  - What stories have you heard about the harms of prostate biopsies?
- What are the harms of prostate cancer treatment?
  - What stories have you heard about the harms of prostate cancer treatment?

**Conversations with providers about PSA**

1. Have you discussed PSA testing with your doctor?

- If YES
  - Tell me about those discussions.
  - What did your provider recommend?
  - Did they explain the reasons for their recommendation?
  - Did they ask for your opinion?
  - How did you react to the recommendation?
  - Did the discussion and recommendation affect your opinion about your provider/ How?
  - About the VA? How?
  - What did you decide to do? Did you have the PSA test?
    - How do you feel about that decision?
    - Is it different from what you decided to do in the past?
- If NO – what is the main reason why you haven’t discussed PSA testing with your doctor?

**Opinions about recommendations to discontinue PSA testing**

1. You may have heard that some doctors now recommend against prostate cancer screening for all men because new research has found that the benefits do not outweigh the harms. What do you think about this recommendation?

- What concerns do you have with this recommendation?
- What are you doing now – are you doing PSA testing?

If yes:

- Imagine your provider told you they didn’t think you should continue having the PSA test.
  - How would you react?
  - Would you be comfortable with stopping testing? Why / why not?
  - What concerns would you have about stopping testing?
- What questions would you want your provider to answer about this?

If No:

- What are the reasons why you are not getting the PSA test?

**Wrap-up**

- Our time is about up here.
- Thank you so much for participating in this interview.
- Your input is very valuable to us.
- We'll use what you shared with us today to develop new educational materials on prostate cancer screening.
